# Supplementary material for: The Prognostic Significance of Puncture Timing to Survival of Arteriovenous Fistulas in Hemodialysis Patients: A Multicenter Retrospective Cohort Study
Source: J Clin Med. 2019 Feb 15;8(2):247. doi: 10.3390/jcm8020247 (PMC6406680; doi:10.3390/jcm8020247)
Supplement: Supplementary file 1 [file jcm-08-00247-s001.pdf]

Supplementary Tables

The Prognostic Significance of Puncture Timing to Survival of Arteriovenous Fistulas in Hemodialysis Patients: A Multicenter Retrospective Cohort Study

Su-Ju Lin[1], Chun-Wu Tung[1,2,3], Yung-Chien Hsu[1,3], Ya-Hsueh Shih[1,3], Yi-Ling Wu[7], Tse-Chih Chou[8], Shu-Chen Chang[7] & Chun-Liang Lin[1,3,4,5,6]

1 Department of Nephrology, Chang Gung Memorial Hospital, Chiayi, Taiwan
2 Graduate Institute of Clinical Medical Sciences, Chang Gung University, Taoyuan, Taiwan
3 Kidney and Diabetic Complications Research Team (KDCRT), Chang Gung Memorial Hospital, Chiayi, Taiwan
4 College of Medicine, Chang Gung University, Taoyuan, Taiwan
5 Kidney Research Center, Chang Gung Memorial Hospital, Taipei, Taiwan
6 Center for Shockwave Medicine and Tissue Engineering, Kaohsiung Chang Gung Memorial Hospital and Chang Gung University College of Medicine, Kaohsiung, Taiwan
7 Research Services Center for Health Information, Chang Gung University, Taoyuan, Taiwan
8 Clinical Informatics and Medical Statistics Research Center, Chang Gung University, Taoyuan, Taiwan

Correspondence and requests for materials should be addressed to C.-L.L. (Professor, Department of Nephrology, Chang-Gung Memorial Hospital, 6 West, Chia-pu Road, Pu-tzu City, Chiayi, Taiwan (R.O.C.); email: linchunliang@cgmh.org.tw; Tel.: +886-5-362-1000, Ext. 2851) or S.-C.C. (email: kathelinchang@gmail.com)

Supplementary Table S1. Characteristics of ESRD patients with AVF (N=26885) grouped by quantile

Demographic characteristics	Functional maturation time[1]				
	Quantile 1 N=6772	Quantile 2 N=6683	Quantile 3 N=6714	Quantile 4 N=6716	P value
Mean ± SD (days)	8.7 ± 4.6	23.7 ± 4.1	60.9 ± 18.4	256.1 ± 223.5	<0.0001
Median (days)	9 ± 8	24 ± 7	59 ± 31	175 ± 160	<0.0001
Men, n (%)	4,166 (61.5)	4,049 (60.6)	4,156 (61.9)	3,502 (52.1)	<0.0001
Age (year), mean ± SD	62.3 ± 13.4	62.6 ± 13.3	63.2 ± 13.1	65 ± 12.8	<0.0001
Comorbidity, n (%)					
HTN	4,864 (71.8)	4,683 (70.1)	4,291 (63.9)	4,619 (68.8)	<0.0001
DM	3,426 (50.6)	3,268 (48.9)	3,012 (44.9)	3,084 (45.9)	<0.0001
MI	240 (3.5)	198 (3)	132 (2)	182 (2.7)	<0.0001
CHF	1,384 (20.4)	1,303 (19.5)	1,123 (16.7)	1,173 (17.5)	<0.0001
PVD	151 (2.2)	149 (2.2)	112 (1.7)	110 (1.6)	0.0083
CVD	629 (9.3)	591 (8.8)	569 (8.5)	693 (10.3)	0.0016
Medication, n (%)					
Aspirin	2,060 (30.4)	1,970 (29.5)	2,126 (31.7)	1,815 (27)	<0.0001
Clopidogrel	784 (11.6)	797 (11.9)	780 (11.6)	827 (12.3)	0.5226
Warfarin	108 (1.6)	97 (1.5)	119 (1.8)	137 (2)	0.0533
Statins	2,361 (34.9)	2,371 (35.5)	2,254 (33.6)	1,803 (26.8)	<0.0001

[1] The time span of functional maturation time in each quantile were ≦16 days, 17-31 days, 32-97 days and ≧98 days respectively.

ESRD, end stage renal disease; AVF, arteriovenous fistula; SD, standard deviation; HTN, hypertension; DM, diabetes mellitus, MI, myocardial infarction; CHF, congestive heart failure; PVD, peripheral vascular disease; CVD, cerebrovascular disease

Supplementary Table S2. Hazard ratios for functional cumulative survival of AVF grouped by quantile

	Event, n (%)	Crude HR (95% CI)	P value	[2]Adjusted HR (95% CI)	P value
Functional maturation time[1]					
Quantile 1	2,076 (30.7)	2.303 (2.135-2.484)	<0.0001	2.295 (2.127-2.476)	<0.0001
Quantile 2	1,963 (29.4)	2.174 (2.014-2.346)	<0.0001	2.172 (2.012-2.345)	<0.0001
Quantile 3	989 (14.7)	Referecnce		Referecnce	
Quantile 4	863 (12.8)	0.921 (0.84-1.009)	0.0762	0.899 (0.82-0.985	0.0226

[1] The time span of functional maturation time in each quantile were ≦16 days, 17-31 days, 32-97 days and ≧98 days respectively.

[2] The model was adjusted for age, gender, hypertension, diabetes mellitus, myocardial infarction, congestive heart failure, peripheral vascular disease, cerebrovascular disease, and use of aspirin, clopidogrel, warfarin, and statins

AVF, arteriovenous fistula; HR, hazard ratios; CI, confidence interval

Supplementary Table S3. Hazard ratios for functional primary patency of AVF grouped by quantile

	Event, n (%)	Crude HR (95% CI)	P value	[2]Adjusted HR (95% CI)	P value
Functional maturation time[1]					
Quantile 1	3,981 (58.8)	1.24 (1.184-1.298)	<0.0001	1.239 (1.184-1.298)	<0.0001
Quantile 2	3,884 (58.1)	1.202 (1.148-1.259)	<0.0001	1.202 (1.148-1.254)	<0.0001
Quantile 3	3,402 (50.7)	Referecnce		Referecnce	
Quantile 4	3,143 (46.8)	0.981 (0.935-1.03)	0.4423	0.977 (0.93-1.026)	0.3444

[1] The time span of functional maturation time in each quantile were ≦16 days, 17-31 days, 32-97 days and ≧98 days respectively.

[2] The model was adjusted for age, gender, hypertension, diabetes mellitus, myocardial infarction, congestive heart failure, peripheral vascular disease, cerebrovascular disease, and use of aspirin, clopidogrel, warfarin, and statins

AVF, arteriovenous fistula; HR, hazard ratios; CI, confidence interval
